# Distinct stress conditions result in aggregation of proteins with similar properties

**DOI:** 10.1038/srep24554

**Published:** 2016-04-18

**Authors:** Alan J. Weids, Sebastian Ibstedt, Markus J. Tamás, Chris M. Grant

**Affiliations:** 1 Faculty of Life Sciences, University of Manchester, Manchester, M13 9PT, UK; 2 Department of Chemistry and Molecular Biology, University of Gothenburg, S-405 30 Gothenburg, Sweden

## Abstract

Protein aggregation is the abnormal association of proteins into larger aggregate structures which tend to be insoluble. This occurs during normal physiological conditions and in response to age or stress-induced protein misfolding and denaturation. In this present study we have defined the range of proteins that aggregate in yeast cells during normal growth and after exposure to stress conditions including an oxidative stress (hydrogen peroxide), a heavy metal stress (arsenite) and an amino acid analogue (azetidine-2-carboxylic acid). Our data indicate that these three stress conditions, which work by distinct mechanisms, promote the aggregation of similar types of proteins probably by lowering the threshold of protein aggregation. The proteins that aggregate during physiological conditions and stress share several features; however, stress conditions shift the criteria for protein aggregation propensity. This suggests that the proteins in aggregates are intrinsically aggregation-prone, rather than being proteins which are affected in a stress-specific manner. We additionally identified significant overlaps between stress aggregating yeast proteins and proteins that aggregate during ageing in yeast and *C. elegans*. We suggest that similar mechanisms may apply in disease- and non-disease settings and that the factors and components that control protein aggregation may be evolutionary conserved.

Protein aggregation is the abnormal association of misfolded proteins into larger, often insoluble structures^1^. Aggregation can be classified into two general categories: amyloid and amorphous. The amyloid state is a highly structured, insoluble, fibrillar deposit, usually consisting of many repeats of the same protein^2^,^3^. This type of aggregation is central in the pathology of many neurodegenerative diseases including Alzheimer’s, Parkinson’s and Huntington’s disease. Amorphous protein aggregation can be best described as the apparently unordered aggregation of proteins, with each individual protein not generally associated with disease when aggregated. Protein misfolding that leads to aggregation can arise as a consequence of age, environmental stress, chemical modifications, destabilising mutations or lack of oligomeric assembly partners^4^,^5^. Newly synthesized proteins appear to be particularly vulnerable to misfolding events and widespread protein aggregation is thought to be toxic, especially when the proteostasis network is compromised^6^,^7^,^8^.

Proteins are highly dynamic molecules, where various modifications or changes in the cellular environment can affect their native conformational fold. Whilst conformational flexibility is required for many proteins to function biologically, aberrant conformations, or misfolding, can lead to protein aggregation^9^. Various stress conditions, such as high temperature, heavy metals and oxidative stress may cause protein misfolding and aggregation by shifting the conformational equilibrium towards more aggregation-prone states where exposed hydrophobic regions of misfolded proteins can interact with other exposed hydrophobic regions leading to aberrant protein-protein interactions^10^,^11^. Therefore, to ensure protein homeostasis, the cell contains an arsenal of molecular chaperones that are able to detect non-native misfolded proteins and act upon them to prevent aggregation or amyloid formation^12^. Generally, it is believed that chaperones are able to target unfolded proteins via recognition of hydrophobic stretches that would otherwise be buried within the native fold and therefore protected from the external environment^9^,^13^. In addition, there are ATP-dependent chaperone classes that are involved in co-translational folding of nascent polypeptides and refolding of proteins (i.e. Hsp70, chaperonins, Hsp90). In the case of Hsp70 s, a co-factor (Hsp40 class chaperones) first assists in recruiting Hsp70 to substrates and then stimulates the ATPase activity of Hsp70 to drive substrate refolding. ATP-independent chaperones, such as the small Hsp class of chaperones, exhibit a ‘holdase’ function by binding to misfolded proteins, preventing their aggregation. Finally, certain chaperones contain a disaggregase function, such as the fungal specific Hsp104^14^. Rather than preventing aggregation, these chaperones act to disassemble protein aggregates.

In a previous study, we isolated aggregation-prone proteins under physiological conditions and arsenite stress and used bioinformatic analyses to identify characteristics that are linked to protein aggregation in living yeast cells. We found that these proteins have high translation rates and are substrates of ribosome-associated Hsp70 chaperones, indicating that they are susceptible to aggregation primarily during translation/folding^15^. The toxic metalloid arsenite promotes protein aggregation by interfering with the folding of nascent polypeptides and by chaperone inhibition^16^. Moreover, bioinformatic analysis of arsenite-induced aggregates suggests that arsenite stress lowers the general threshold for protein aggregation^15^,^16^. Together, these studies suggest that protein aggregation is a normal physiological event, but conditions which perturb cellular homeostasis can increase the burden of protein aggregation.

In this current study, we have extended our analysis of protein aggregation to include two additional stress conditions – azetidine-2-carboxylic acid (AZC) and hydrogen peroxide (H_2_O_2_) – that promote misfolding and aggregation through different mechanisms. AZC is a proline analogue which is competitively incorporated into proteins in place of proline^17^. AZC incorporation alters the conformation of the polypeptide backbone, resulting in widespread protein misfolding and aggregation^6^. H_2_O_2_ is a ubiquitous stress agent that is formed as a byproduct of aerobic respiration and following exposure to diverse biological and environmental factors. H_2_O_2_ gives rise to oxidative stress in cells which may in turn damage proteins and promote protein aggregation^18^,^19^,^20^. Using computational approaches, we characterized the proteins that aggregate following AZC and H_2_O_2_ stress and compared them with proteins which aggregate during arsenite stress or during physiological conditions. Our data indicate that the three stress conditions, which work by distinct mechanisms, promote the aggregation of similar types of proteins, probably by lowering the threshold of protein aggregation. This suggests that the proteins in aggregates are intrinsically aggregation-prone, rather than being proteins which are affected in a stress-specific manner. Most proteins are susceptible for aggregation during synthesis/folding. In addition, certain proteins may aggregate post-translationally due to an imbalance between abundance and solubility.

## Results

### Identification of stress-induced protein aggregates in yeast

**Figure 1:**
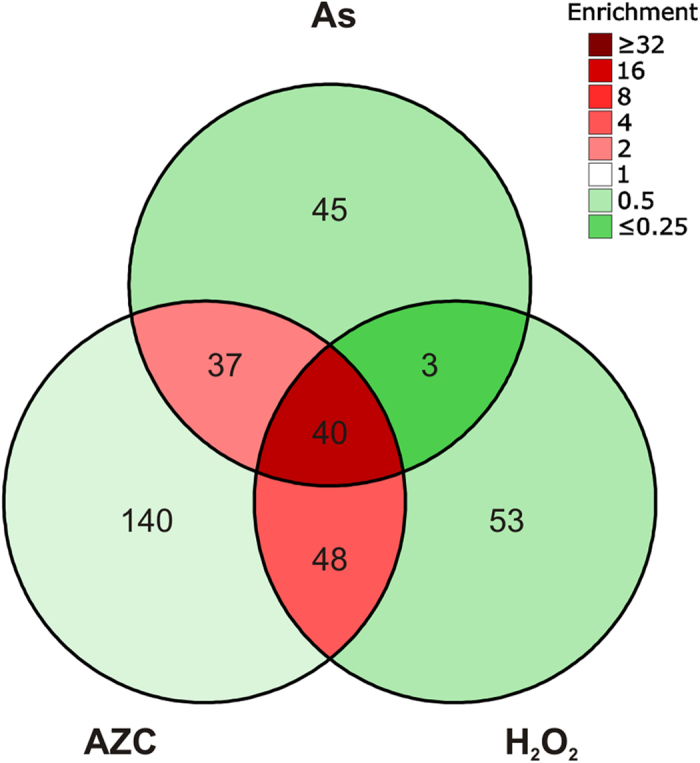
Proteins that aggregate during three distinct stress conditions in yeast. Number of proteins identified in aggregate fractions during As, AZC and H_2_O_2_ stress. For comparative analyses, the aggregated proteins were partitioned into non-overlapping datasets; 45 proteins uniquely identified in the As-set, 140 proteins within the AZC-set, 53 proteins within the H_2_O_2_-set, and a stress-set (Common-set) which contains 128 proteins that aggregate in at least two of the three stress conditions. The colours correspond to enrichment, taking the number of identifiable proteins into account.

To extend our previous analyses of protein aggregation^15^,^16^, protein aggregates were isolated and identified following H_2_O_2_ and AZC stress. Insoluble protein aggregates were prepared as previously described^21^,^22^ and identified using mass spectrometry following three independent experiments (see Materials and Methods). The H_2_O_2_-set and AZC-set were compared with our previously identified set of proteins which aggregate following arsenite stress (As-set)^15^. We noted that a considerable fraction of the proteins (~35%) aggregated in response to more than one stress condition (Fig. 1). Therefore, to facilitate a true comparative analysis of the stress-induced protein aggregates, we partitioned the identified proteins into non-overlapping datasets; 45 proteins uniquely identified in the As-set, 140 proteins within the AZC-set, 53 proteins within the H_2_O_2_-set, and a stress-set (Common-set) which contains 128 proteins that aggregate in at least two of the three stress conditions (Fig. 1).

### Functional analysis of aggregation-prone proteins

**Figure 2:**
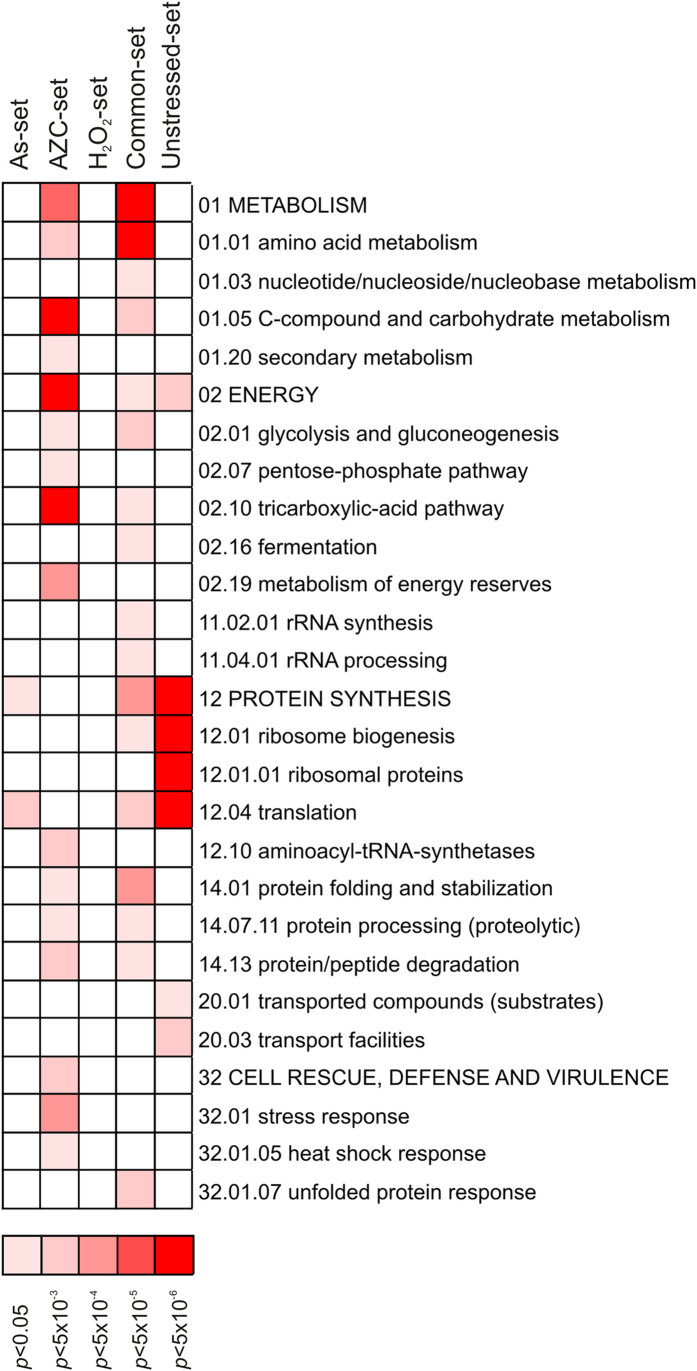
Functional analysis of aggregation-prone proteins. Significantly enriched functional categories within the data-sets were determined using FunCat (FDR < 5%). Results are ordered on MIPS category classification numbers and overarching categories are in capitals.

We first performed gene ontology analysis to examine what functional categories of proteins are enriched in the aggregate fractions following the three distinct stress conditions. For this and all subsequent analyses, our stress datasets were compared with an unstressed dataset, which is comprised of proteins that aggregate only under normal physiological conditions (Unstressed-set). Significantly enriched (5% FDR) functional categories were determined within the datasets using the MIPS Functional Catalogue^23^. As we previously described^15^, factors involved in protein synthesis including ribosomal and translation related proteins are strongly enriched within proteins that aggregate in the absence of stress (Fig. 2; Unstressed dataset). Additionally, proteins involved in energy and transport functions are enriched within these aggregates. More functional categories were enriched in the Common-set compared with the Unstressed-set; these include many protein synthesis related functions, as well as proteins involved in metabolism and energy related processes. There was also enrichment for proteins involved in protein folding, stabilisation and processing, as well as components of the unfolded protein response. These latter classes of proteins would be expected to constitute part of the cellular response to protein misfolding and aggregation. Stress-specific differences were found in the functional classes that are enriched under different stress conditions. The As-specific set was significantly enriched for proteins related to protein synthesis and translation (Fig. 2), in line with the notion that arsenite interferes with folding of nascent polypeptides^15^,^16^. The AZC-specific set was enriched in a large number of categories, including metabolism, energy and protein synthesis-related functions, as well as cell rescue and defence proteins including a number of chaperones (Fig. 2). The large number of functional groups enriched in the AZC-specific data-set may be a reflection of its mode of action, as AZC will affect all proline-containing proteins. In contrast to the other sets, no functional groups were significantly enriched within the H_2_O_2_-specific set. Taken together, these data indicate that protein aggregates isolated from different conditions show enrichment for a number of similar functional categories.

### Stress conditions shift the physicochemical criteria for aggregation propensity

**Figure 3:**
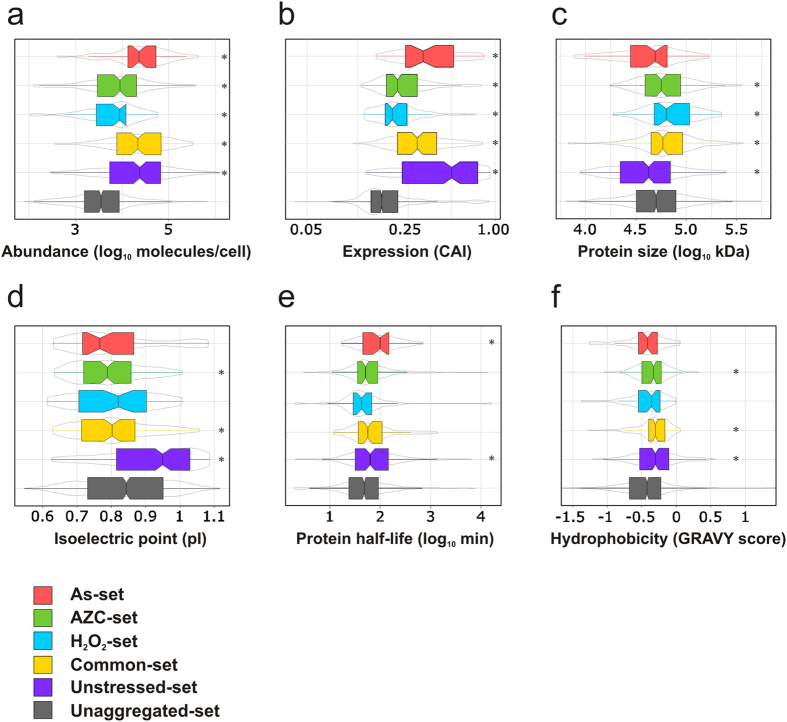
Properties of aggregation-prone proteins. (**a**) Abundance. The abundance of proteins (molecules/cell) in each set during non-stress conditions^43^ is plotted. (**b**) Expression levels. The codon adaptation index (CAI) is an indicator of gene expression level and the CAI for proteins in each set is plotted. (**c**) Protein size. The molecular weights (kDa) of proteins in each set is plotted. (**d**) Isoelectric point (pI). The pI values of the proteins in each set are shown. (**e**) Protein half-lives. The half-lives of proteins in each set under non-stress conditions^25^ is plotted. (**f**) Hydrophobicity. The GRAVY scores of the proteins in each set is plotted. Statistical analyses were performed as described in Methods, and *indicates a significant difference (*p *< 0.05) compared to the Unaggregated set.

Our previous observations suggested that arsenite stress lowers the overall threshold for protein aggregation^15^. To determine whether this is also true for other stresses that promote protein aggregation, we assessed a number of physicochemical properties of the proteins within our datasets. For comparison, a list of yeast proteins detectable by mass spectrometry in logarithmically growing cells was used to represent the properties of unaggregated proteins^24^. Aggregated proteins in the Unstressed-set are more abundant (*i.e*. present in more molecules/cell), more highly expressed (indicated by a high codon adaptation index (CAI)), smaller in size (*i.e*. lower molecular weight (MW)), and have a higher isoelectric point (pI) than proteins in the Unaggregated set (Fig. 3a–d). Similar to the Unstressed-set, highly expressed and abundant proteins are significantly enriched in the aggregate fractions following all three stress conditions (Fig. 3a,b). However, the proteins which aggregate under stress conditions have considerably lower abundance and expression levels compared with the Unstressed-set (Fig. 3a,b). Proteins that aggregate following the three stress conditions also have a lower pI than the proteins in the Unstressed set; their pIs are similar to the Unaggregated-set (H_2_O_2_-set and As-set) or lower (Common-set and AZC-set) (Fig. 3d). Stress-specific differences are also found when the sizes of the aggregated proteins are compared (Fig. 3c). Whilst the proteins in the Unstressed-set are generally smaller in size than the unaggregated set, proteins which aggregate in the Common-set, H_2_O_2_-set and AZC-set are significantly larger than those in the Unstressed-set. Thus, the proteins that aggregate following stress conditions have lower expression levels, are less abundant, are more acidic and are larger than the proteins which aggregate during physiological non-stress conditions.

Given the increased abundance of aggregated proteins during both unstressed and stressed conditions compared to the Unaggregated set, we examined whether this correlates with protein stability. For this analysis, we used a data-set of protein half-lives as determined by measuring protein abundance over time after inhibition of protein biosynthesis^25^. The proteins which aggregate under non-stress (Unstressed-set) or arsenite stress (As-set) have on the average a longer half-life than the proteins in the Unaggregated set (Fig. 3e). However, no significant differences in protein stability are observed for the proteins in the Common-, H_2_O_2_- or AZC-set compared with the Unaggregated set (Fig. 3e). Thus, increased protein stability does not appear to account for the increased abundance of the proteins which tend to aggregate.

Finally, we investigated the hydrophobicity (GRAVY score) of the aggregated proteins. Proteins in the Unstressed-set, the Common-set and the AZC-set are generally more hydrophobic than proteins in the Unaggregated-set (Fig. 3f). However, the proteins that aggregate under stress are less hydrophobic than those that aggregate in the absence of stress. We conclude that proteins that aggregate during physiological conditions and stress share several features, and that stress conditions shift the criteria for protein aggregation propensity.

### Amino acid composition of protein aggregates

**Figure 4:**
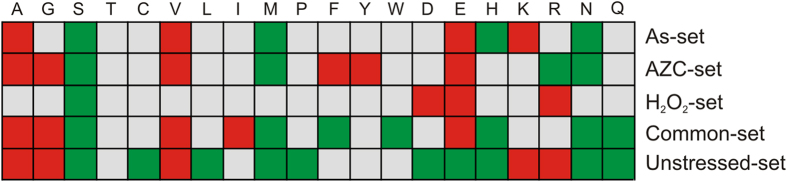
Amino acid composition of aggregation-prone proteins. For each amino acid, the average percentage content was calculated from the proteins in each set and compared to the average amino acid content in the Unaggregated set. Red indicates a significant (*p *> 0.05) enrichment whereas green indicates a significant (*p *> 0.05) depletion compared to the Unaggregated set. Grey indicates no significant difference.

We next examined the relative amino acid content of the proteins enriched in our aggregate fractions. Proteins in the Unstressed-set are enriched in aliphatic amino acids including Ala, Gly and Val compared with the Unaggregated-set (Fig. 4), in agreement with their hydrophobic character (Fig. 3f). Furthermore, basic amino acids (Lys and Arg) are strongly enriched, whereas acidic amino acids (Asp, Glu) are underrepresented in the Unstressed-set (Fig. 4), which accounts for their higher pI compared with the Unaggregated-set (Fig. 3d). A number of amino acids are underrepresented in the Unstressed-set including Pro, Ser, His and sulphur-containing amino acids (Met, Cys) (Fig. 4). We also found that Gln and Asn are underrepresented in the Unstressed-set suggesting that the proteins in these aggregates are distinct from the well-known amyloid forming proteins. These amino acids are normally thought to underlie amyloid formation and have been linked to prion formation in yeast and mammalian neurological disorders including Huntington’s disease^26^.

The stress-aggregated proteins also show differences in their amino acid content compared to the Unaggregated protein set (Fig. 4). However, we did not observe any strong correlations with amino acids that are known to be targeted by the different stress conditions. For example, the AZC-set is not enriched in proline residues suggesting that they are not simply proteins where excess AZC is incorporated. Arsenite and H_2_O_2_ are known to target cysteine-containing proteins as part of their mode of toxicity, but the relative cysteine content of proteins within the As- and H_2_O_2_-set is not enriched (Fig. 4). This suggests that the proteins identified during stress conditions represent intrinsically aggregation-prone proteins. In agreement with this hypothesis, and similar to the Unstressed-set, the Common-set is enriched for hydrophobic proteins (Fig. 3f) and aliphatic amino acids including Ala, Gly, Ile, Val (Fig. 4). However, stress-specific differences are seen in the content of hydrophobic proteins (and aliphatic amino acids) as the As- and H_2_O_2_-specific sets are not significantly enriched for hydrophobic proteins compared with the Unaggregated-set (Fig. 3f). We note though that some aliphatic amino acids (Ala, Val) are enriched in the As-set (Fig. 4), indicating a weak correlation between hydrophobicity and As-specific aggregation. A number of amino acids that are underrepresented in the Unstressed-set are also underrepresented in the Common-set including Asn, Gln, Ser, His and Met. Thus, while a high content of aliphatic amino acids is positively correlated with protein aggregation, a high content of Asn, Gln, Ser, His and Met might be negatively correlated with physiological and stress-induced aggregation.

### Proteins are primarily susceptible for aggregation during translation/folding

**Figure 5:**
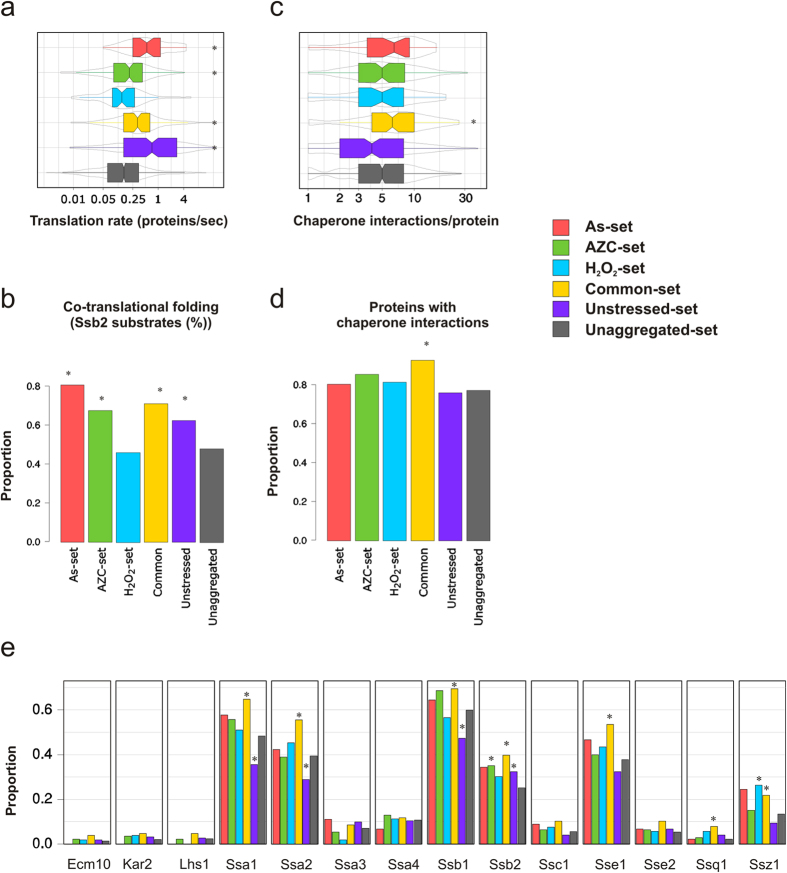
Proteins are vulnerable for aggregation during synthesis/folding. (**a**) Translation rate. Estimated translation rates^27^ per protein in each set is shown. (**b**) Co-translational folding. Bars indicate the proportion of proteins in each set that are co-translational substrates of Ssb2^28^. **(c**) Chaperone interactions. The number of chaperone interactions per protein in each set is plotted. **(d**) Chaperone interactions. The proportion of proteins in each set with at least one chaperone interaction is plotted. (**e**) Interactions with Hsp70 chaperones. The proportion of proteins in each set that interact with a specific Hsp70 chaperone is plotted. Statistical analyses were performed as described in Methods, and *indicates a significant difference (*p *< 0.05) compared to the Unaggregated set.

Given that the aggregated proteins identified under both non-stress and stress conditions are highly expressed and abundant, we compared their translation rates with unaggregated proteins. For this analysis we compared the aggregated proteins with a genome-wide estimate of translation rates^27^. Proteins that aggregate under unstressed conditions are significantly enriched for proteins which show high rates of mRNA translation compared with unaggregated proteins (Fig. 5a). Similarly, in agreement with their higher protein abundances and expression levels (Fig. 3a,b), translation rates are also significantly increased in the Common-set as well as the As- and AZC-specific sets (Fig. 5A). Nevertheless, these proteins have considerably lower translation rates compared with the proteins in the Unstressed-set (Fig. 5a). In contrast, translation rates are not increased for the H_2_O_2_-set despite their enrichment for higher abundance and expression proteins (Fig. 5a).

It is now well established that many proteins are subject to co-translational folding, predominantly mediated by Hsp70 family chaperones. For example, Ssb1 and Ssb2 are ribosome-associated chaperones that are important in the folding of nascent polypeptide chains. We therefore examined whether aggregated proteins are enriched for co-translational substrates of Ssb chaperones using available Ssb2 data^28^. In accordance with their high translation rates, proteins in the As-, AZC-, Common- and Unstressed-set are significantly enriched in proteins that are co-translational Ssb2 substrates compared to Unaggregated proteins (Fig. 5B). In contrast, the H_2_O_2_-specific set is not enriched for co-translational Ssb2 substrates. Taken together, these findings suggest that proteins are primarily susceptible for aggregation during translation/folding, both during physiological conditions and during stress. The exception appears to be H_2_O_2_ (see Discussion).

### Proteins which aggregate under stress conditions are enriched for chaperone interactions

We previously reported that proteins which aggregate following arsenite stress are significantly enriched in proteins with more chaperone interactions per protein compared with proteins in the proteome^15^. This analysis was performed using a chaperone-protein interaction atlas for 63 chaperones present in yeast^29^. When this analysis was repeated here using the non-overlapping datasets, we found that proteins that aggregate following stress conditions (Common-set) are significantly enriched for multiple chaperone interactions (Fig. 5c,d). The As-, H_2_O_2_-, and AZC-specific sets and the Unstressed-set were not enriched, nor depleted, for chaperone-interacting proteins compared to the Unaggregated-set.

Due to the importance of Hsp70 s in co-translational folding of nascent polypeptides and refolding of proteins, we next asked whether the aggregated protein sets are enriched for interactions with specific members of the Hsp70 family. For this, we chose seven cytoplasmic (Ssa1–4, Sse1–2, Ssz1), two ribosome-associated (Ssb1, Ssb2), two ER (Kar2, Lhs1) and three mitochondrial (Ssc1, Ssq1, Ecm10) chaperones (Fig. 5e) from the chaperone-protein interaction atlas^29^. The Common-set is enriched for Ssa1, Ssa2, Ssb1, Ssb2, Sse1, Ssq1, and Ssz1 interacting proteins compared to the Unaggregated-set (Fig. 5E). The Unstressed- and AZC-specific sets were both enriched for Ssb2 interactions whilst the H_2_O_2_-set was enriched for Ssz1 interactions. The Unstressed-set was also significantly underrepresented for Ssa1, Ssa2, and Ssb1 interactions (Fig. 5e), possibly because the Unstressed-set contains many ribosomal proteins that require specialized chaperones for proper folding^30^. Thus, Hsp70 chaperone-interacting proteins therefore appear to be significantly enriched in stress-induced aggregates compared with unstressed protein aggregates. Few stress-specific interactions were observed, except for the ribosome associated Ssz1-interacting proteins which is enriched in the H_2_O_2_-set. None of the aggregated protein sets were significantly enriched for interactions with Ecm10, Kar2, Lhs1, Ssa3, Ssa4, Ssc1 and Sse2 compared with the Unaggregated set. Taken together, stress-aggregated proteins are enriched for multiple chaperone interactions.

### Molecular chaperones are present within protein aggregates

**Figure 6:**
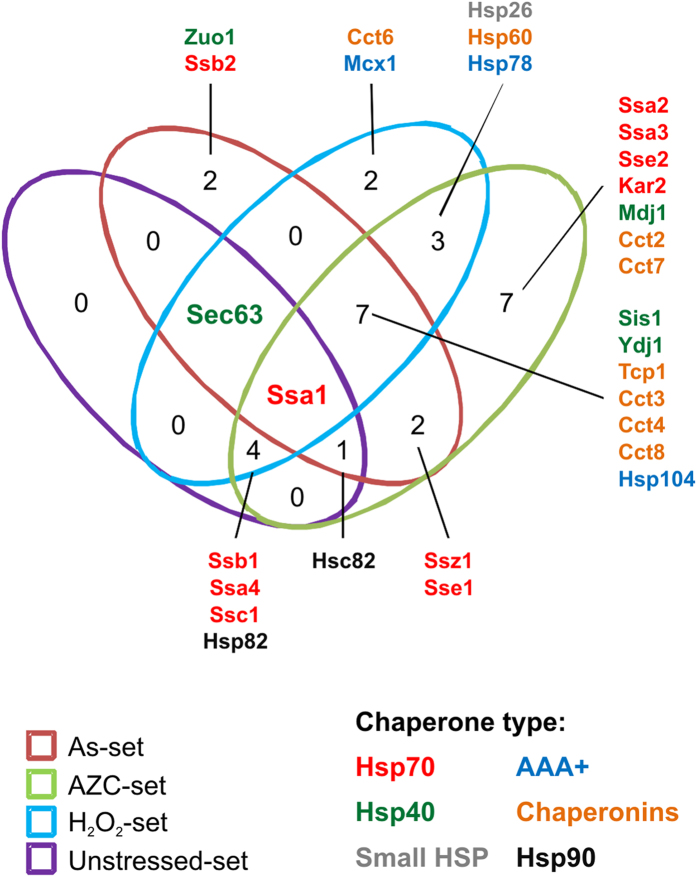
Molecular chaperones present in the aggregates. Molecular chaperones were identified within the datasets and the overlap between the datasets is presented. The chaperone types are indicated by colour of the text.

Given that proteins with multiple chaperone interactions are enriched within stress-aggregated sets, we examined whether chaperones were isolated in our aggregate fractions. It should be emphasised that our analysis does not allow us to differentiate between chaperones which are functional components of the aggregates, versus chaperones which are themselves aggregation-prone. Of the 63 known chaperones in *S. cerevisiae*^29^, we identified 30 chaperones distributed between all the datasets: 11 Hsp70 s, five Hsp40 s, seven chaperonin subunits, three AAA+ family members, two Hsp90 s, one Hsp60 and one small Hsp chaperone (Fig. 6). A total of seven chaperones are present in the Unstressed-set: Ssb1, Ssa1, Ssa4, Ssc1, Hsp82, Hsc82 and Sec63. We also identified chaperones within the non-overlapping stress sets. The Common-set included 19 chaperones spanning all six chaperone classes (Fig. 6). All of these chaperones except Ssc1, Hsp78, Hsp60 and Sec63, are present in the cytoplasm. Their inclusion within the Common-set may indicate that these chaperones are part of a general cytoplasmic response to stress and that they are associated with their client proteins. Seven chaperones were identified within the AZC-specific set; four of these (Ssa2, Ssa3, Sse2, Kar2) belong to the Hsp70 family, whilst one (Mdj1) is a known co-factor of proteins of the Hsp70 family (Fig. 6). This may be indicative of the mechanism of action of AZC, as Hsp70 family proteins are important for co-translational folding. Two chaperones were present in the As-specific set (Zuo1 and Ssb2), and both are ribosome-associated chaperones (Fig. 6). This is in agreement with the notion that arsenite primarily targets nascent proteins for aggregation. The H_2_O_2_-specific set contained two chaperones (Cct6 and Mcx1) but it is currently unclear how these chaperones relate to H_2_O_2_’s mode of action. The H_2_O_2_-set was enriched for proteins that interact with Ssz1 – however, this chaperone is absent from the aggregates themselves. This may suggest that H_2_O_2_ might inhibit Ssz1 function.

### Age-related protein aggregation may be promoted by multiple stresses

**Figure 7:**
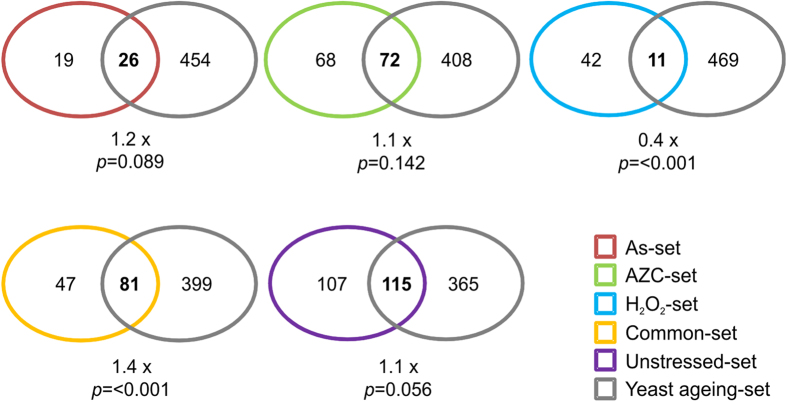
Overlap between age-dependent and stress-dependent aggregation. Yeast proteins within our datasets were compared with proteins that were found to aggregate during postmitotic ageing in yeast^31^. Significance of the overlap was determined by a hypergeometric test and the fold difference over the expected overlap value is displayed.

Protein aggregation is a hallmark of many ageing related diseases. A recent study identified 480 proteins that aggregate during postmitotic ageing in yeast^31^. When we compared these proteins with our datasets we found no significant overlap with our Unstressed-set. This makes sense given that our unstressed proteins were identified in exponential phase yeast cells rather than in an aged population. Interestingly, a significant overlap (1.4-fold, *p *= <0.001) was found between the ageing dataset and the Common-set, although there was no significant overlap for any single stress-specific condition (Fig. 7). Therefore, it appears that proteins which generally aggregate in response to stress are also likely to aggregate in aged yeast cells.

### Aggregation prone proteins are conserved from yeast to *C. elegans*

**Figure 8:**
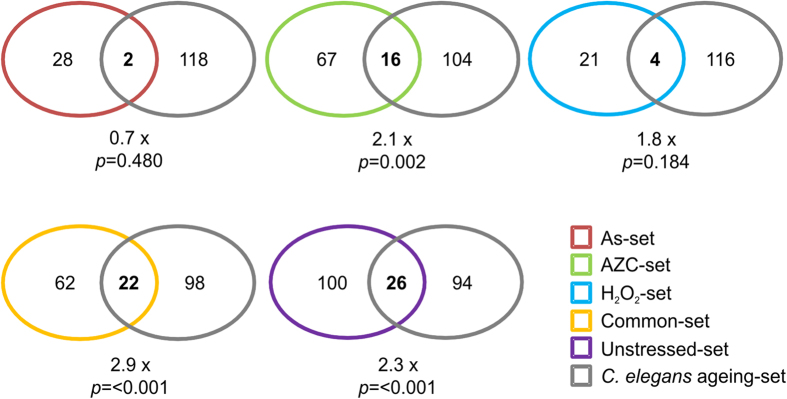
Overlap between stress-dependent aggregation in yeast and age-dependent aggregation in *C. elegans*. Yeast proteins within our datasets were converted (if existent) to their orthologous *C. elegans* protein(s) and compared with the *C. elegans* proteins with a yeast orthologue from the 461 proteins isolated by^32^. Significance of the overlap was determined by a hypergeometric test and the fold difference over the expected overlap value is displayed.

Finally, we wanted to compare our datasets with proteins that aggregate in another organism. A previous study identified 461 proteins in aged *C. elegans* protein aggregates^32^. Of these 461 proteins, 120 have a recognisable yeast orthologue which we used for comparative analysis with our datasets (referred to as CE-set). Within our protein aggregate datasets, we found that 126 (Unstressed-set), 30 (As-set), 25 (H_2_O_2_-set), 83 (AZC-set) and 84 (Common-set) proteins have recognisable *C. elegans* orthologues. Our analysis revealed a significant overlap between the yeast stress datasets (Common-set) and the CE-set. Overall, 69 (57.5%) of the proteins in our stress dataset are present in the CE-set (Fig. 8). 26 of the 126 orthologous proteins in the Unstressed-set overlap with the CE-set (22% of CE-set; 2.3 fold above the expected overlap; *p *= <0.001) (Fig. 8). Of the 26 overlapping proteins, 18 are ribosomal proteins and two are Hsp70 family chaperones. Significant overlaps are also found with the AZC- and Common-sets. Notable proteins, in which their presence in aggregates is conserved between our stress datasets and the CE-set, include components of the essential chaperonin Ctt ring complex. Subunits, Tcp1, Cct3, Cct4 and Cct8 are present in all our stress sets and have like-for-like orthologues in *C .elegans*, which are present in the CE-set. Another essential protein, Cdc48, is also conserved in all stress induced and CE-set aggregates. *C. elegans* has two orthologous of Cdc48 (cdc-48.1 and cdc-48.2), of which both forms are present in CE-set.

## Discussion

In this study, we used computational approaches to characterize proteins that aggregate in the absence of stress and under three distinct stress conditions (arsenite, H_2_O_2_ and AZC). We found that proteins that aggregate during physiological conditions and during stress are generally more abundant, highly expressed and translated at faster rates, when compared to the wider proteome. This extends our previous finding that high protein abundance positively correlates with an increased aggregation propensity^15^. Previous studies have predicted that aggregation propensity is not correlated with protein expression and abundance, *i.e*. highly expressed and abundant proteins have evolved to be both highly soluble and resistant to aggregation^33^,^34^,^35^,^36^. However, it was noted that, although proteins are expressed at a level to allow functionality in balance with their intrinsic aggregation propensity, there is almost no flexibility with this equilibrium^35^. Hence, any factors that would decrease solubility or increase the concentration of a protein would result in unavoidable aggregation^35^. Indeed, a recent study has identified a number of proteins which are maintained at a high concentration relative to their solubility^37^. It was proposed that these ‘supersaturated’ proteins are highly dependent on the proteostasis network to maintain their solubility, and that any perturbations in this network will shift supersaturated proteins towards aggregation^37^. For example, the proteostasis network is believed to decline during ageing and protein aggregates isolated from aged *C. elegans* were found to be enriched for supersaturated proteins^1^,^32^,^37^,^38^. Protein abundance alone can therefore be a good indicator of protein aggregation *in vivo*. Our results are in striking agreement with this hypothesis; we found that proteins that aggregate during physiological unstressed conditions and during stress are generally more abundant, highly expressed and translated at faster rates, when compared to the wider proteome.

Our data indicate that the three stress conditions, which each work by distinct mechanisms, promote the aggregation of similar types of proteins; these proteins are abundant, highly expressed, and translated at high rates. Stress-aggregated proteins tend to be somewhat hydrophobic, more acidic and larger in size compared to unaggregated proteins. As they share many features, these proteins are likely intrinsically aggregation-prone, rather than being proteins which are affected in a stress-specific manner. Our current study further revealed that cellular stress conditions act to lower the threshold of protein aggregation. Notably, the abundance, expression levels and translation rates of the stress-aggregated proteins were not as high as for the proteins that aggregate during physiological conditions. Likewise, proteins that aggregate during environmental stress were less hydrophobic than those aggregating during physiological conditions. Thus, proteins that are normally not susceptible to aggregation become aggregation-prone during stress.

A large fraction of the aggregated proteins are co-translational substrates of ribosome-associated Ssb2. Moreover, the stress aggregated proteins (Common-set) are enriched for chaperone interactions including several Hsp70-interacting proteins (Ssb1, Ssb2, Ssa1, Ssa2, Sse1, Ssq1, Ssz1). Thus, these proteins appear susceptible for aggregation primarily during synthesis/folding, indicating that stress conditions promote co-translational aggregation. The exception appears to be H_2_O_2_ which may be because oxidative stress strongly inhibits translation^39^ and hence translation rate is no longer differentiated in the proteins which aggregate following H_2_O_2_ stress. Alternatively, H_2_O_2_-stress may additionally target native proteins for aggregation. The unstressed dataset is translated at higher rates than the unaggregated and stressed datasets and is also enriched for proteins that interact with ribosome-bound Ssb2 indicating co-translational aggregation. However, we found that the Unstressed set is generally depleted of Hsp70 chaperones (Ssb1-, Ssa1- and Ssa2-interacting), but not for chaperones in general. Thus, physiological aggregates which form under non-stressed conditions may also contain a fraction of proteins that are less prone to co-translational aggregation. In line with the supersaturated protein theory, these proteins may aggregate when their abundance exceeds their solubility.

It is well established that certain amino acids influence protein aggregation, and that proteins with solvent exposed stretches of high hydrophobicity and a low net charge are aggregation-prone. Indeed, aliphatic amino acids along with basic amino acids were over-represented in physiological aggregates (Unstressed set). Aliphatic amino acids were also enriched within stress-induced aggregates. However, in contrast to the Unstressed-set, the Common-set is enriched for acidic amino acids (Asp and Glu) rather than basic amino acids. Thus, proteins that aggregate under stress conditions are generally acidic whilst proteins that aggregate under physiological conditions are generally basic. It has been proposed that hydrophobic and electrostatic forces that promote the formation of functional protein complexes can also cause abnormal protein-protein interactions^40^. Indeed, aggregation prone regions enriched in aliphatic amino acids appear to be important in protein-protein interactions^40^,^41^. Interestingly, positively charged residues (including Arg and Lys) that flank aggregation prone regions strongly oppose close packing and aggregation. Hence, it is thought that these aggregation resistant flanks provide specificity to hydrophobic-mediated protein-protein interactions. In contrast, Glu and Asp appear to be ineffective in disrupting aggregation when compared to Lys and Arg^41^. A large fraction of the aggregated proteins identified in this study are complex forming proteins (e.g. ribosomal proteins). Moreover, we previously showed that proteins aggregating during physiological conditions and arsenite stress are engaged in a significantly higher number of protein-protein interactions per protein than the proteins in the proteome^15^. Thus, our data are in agreement with the notion that protein complex formation and aggregation may be governed by similar principles.

We found a significant overlap between proteins that aggregate during yeast ageing and during stress (Common-set). Our analysis also revealed significant overlap between stress aggregating yeast proteins (Common-set) and proteins that aggregate in ageing *C. elegans*. There is also an overlap between ageing-dependent protein aggregation in *C. elegans* and disease-dependent aggregation in mammals^32^. Several aggregation-prone yeast proteins have human homologues that are implicated in protein misfolding diseases. Thus, similar mechanisms may apply in disease- and non-disease settings and the factors and components that control protein aggregation may be evolutionary conserved.

## Methods

### Analysis of insoluble protein aggregates

Following SD (untreated), AZC (5 mM) or H_2_O_2_ (1 mM) treatment for 2 hours, insoluble protein aggregates were isolated as previously described^16^,^22^. Insoluble protein extracts were separated by reducing SDS/PAGE (12% gels) and visualized by silver staining with the Bio-Rad silver stain plus kit. Aggregated proteins were identified by mass spectrometry (performed by the Biomolecular Analysis Core Facility, Faculty of Life Sciences, The University of Manchester) in triplicate for each condition. For protein identification, protein samples were run a short distance into SDS-PAGE gels and stained using colloidal Coomassie blue (Sigma). Total proteins were excised, trypsin digested, and identified using liquid chromatography-mass spectrometry (LC-MS). Proteins were identified using the Mascot mass fingerprinting programme (www.matrixscience.com) to search the NCBInr and Swissprot databases. Final datasets for each condition were determined by selecting proteins that were identified in at least two of the three replicates.

### Statistical analyses

Datasets for each condition were assessed for functional enrichment (*p*-value > 0.01; 0.05 FDR) of functional categories (MIPS database) using FunCat (available at http://www.helmholtz-muenchen.de/en/ibis. Protein abundance data was retrieved from the PaxDB integrated dataset (available at http://pax-db.org). The proteins that aggregate in aged-yeast or *C. elegans* were retrieved from published sources^31^,^42^ and statistical significance of the overlap was evaluated with a hypergeometric test. Enrichment and significance of aggregation propensity under one, two or three stress conditions were estimated with Monte Carlo simulations, using as background a list of proteins which are expected to be identifiable with LC-MS^24^. Venn diagrams and visualization of the distribution of protein hits between conditions were made using Venny (http://bioinfogp.cnb.csic.es/tools/venny/). Physicochemical data, translation rates and chaperone interactions were evaluated with pair wise Mann-Whitney-Wilcoxon U-tests, followed by Holm-Bonferroni adjustments to avoid inflation of Type I error rate. Amino acid content was assessed statistically with Mann-Whitney-Wilcoxon U-test, and *p*-values were filtered on 0.05 FDR.

## Additional Information

**How to cite this article**: Weids, A. J. *et al*. Distinct stress conditions result in aggregation of proteins with similar properties. *Sci. Rep*. **6**, 24554; doi: 10.1038/srep24554 (2016).

## References

[b1] Hipp M. S., Park S. H. & Hartl F. U. Proteostasis impairment in protein-misfolding and -aggregation diseases. Trends Cell Biol 24, 506–514 (2014). 24946960 10.1016/j.tcb.2014.05.003

[b2] Greenwald J. & Riek R. Biology of amyloid: structure, function, and regulation. Structure 18, 1244–1260 (2010). 20947013 10.1016/j.str.2010.08.009

[b3] Tipping K. W., van Oosten-Hawle P., Hewitt E. W. & Radford S. E. Amyloid Fibres: Inert End-Stage Aggregates or Key Players in Disease? Trends Biochem Sci 40, 719–727 (2015). 26541462 10.1016/j.tibs.2015.10.002 PMC7617725

[b4] Vendruscolo M. Proteome folding and aggregation. Curr Opin Struct Biol 22, 138–143 (2012). 22317916 10.1016/j.sbi.2012.01.005

[b5] O’Connell J. D., Zhao A., Ellington A. D. & Marcotte E. M. Dynamic reorganization of metabolic enzymes into intracellular bodies. Annu Rev Cell Dev Biol 28, 89–111 (2012). 23057741 10.1146/annurev-cellbio-101011-155841 PMC4089986

[b6] Weids A. J. & Grant C. M. The yeast peroxiredoxin Tsa1 protects against protein-aggregate-induced oxidative stress. J Cell Sci 127, 1327–1335 (2014). 24424024 10.1242/jcs.144022 PMC3953820

[b7] Winkler J. . Quantitative and spatio-temporal features of protein aggregation in Escherichia coli and consequences on protein quality control and cellular ageing. EMBO J 29, 910–923 (2010). 20094032 10.1038/emboj.2009.412 PMC2837176

[b8] Koplin A. . A dual function for chaperones SSB-RAC and the NAC nascent polypeptide-associated complex on ribosomes. J Cell Biol 189, 57–68 (2010). 20368618 10.1083/jcb.200910074 PMC2854369

[b9] Hartl F. U., Bracher A. & Hayer-Hartl M. Molecular chaperones in protein folding and proteostasis. Nature 475, 324–332 (2011). 21776078 10.1038/nature10317

[b10] Vabulas R. M., Raychaudhuri S., Hayer-Hartl M. & Hartl F. U. Protein folding in the cytoplasm and the heat shock response. Cold Spring Harb Perspect Biol 2, a004390 (2010). 21123396 10.1101/cshperspect.a004390 PMC2982175

[b11] Tamas M. J., Sharma S. K., Ibstedt S., Jacobson T. & Christen P. Heavy metals and metalloids as a cause for protein misfolding and aggregation. Biomolecules 4, 252–267 (2014). 24970215 10.3390/biom4010252 PMC4030994

[b12] Kim Y. E., Hipp M. S., Bracher A., Hayer-Hartl M. & Hartl F. U. Molecular chaperone functions in protein folding and proteostasis. Annu Rev Biochem 82, 323–355 (2013). 23746257 10.1146/annurev-biochem-060208-092442

[b13] Flynn G. C., Pohl J., Flocco M. T. & Rothman J. E. Peptide-binding specificity of the molecular chaperone BiP. Nature 353, 726–730 (1991). 1834945 10.1038/353726a0

[b14] Verghese J., Abrams J., Wang Y. & Morano K. A. Biology of the heat shock response and protein chaperones: budding yeast (Saccharomyces cerevisiae) as a model system. Microbiol Mol Biol Rev 76, 115–158 (2012). 22688810 10.1128/MMBR.05018-11 PMC3372250

[b15] Ibstedt S., Sideri T. C., Grant C. M. & Tamas M. J. Global analysis of protein aggregation in yeast during physiological conditions and arsenite stress. Biology open 3, 913–923 (2014). 25217615 10.1242/bio.20148938 PMC4197440

[b16] Jacobson T. . Arsenite interferes with protein folding and triggers formation of protein aggregates in yeast. J Cell Sci 125, 5073–5083 (2012). 22946053 10.1242/jcs.107029

[b17] Trotter E. W., Berenfeld L., Krause S. A., Petsko G. A. & Gray J. V. Protein misfolding and temperature up-shift cause G1 arrest via a common mechanism dependent on heat shock factor in Saccharomycescerevisiae. Proc Natl Acad Sci USA 98, 7313–7318 (2001). 11416208 10.1073/pnas.121172998 PMC34665

[b18] Hohn A., Jung T. & Grune T. Pathophysiological importance of aggregated damaged proteins. Free Radic Biol Med 71, 70–89 (2014). 24632383 10.1016/j.freeradbiomed.2014.02.028

[b19] Dukan S. . Protein oxidation in response to increased transcriptional or translational errors. Proc Natl Acad Sci USA 97, 5746–5749 (2000). 10811907 10.1073/pnas.100422497 PMC18504

[b20] Ling J. & Soll D. Severe oxidative stress induces protein mistranslation through impairment of an aminoacyl-tRNA synthetase editing site. Proc Natl Acad Sci USA 107, 4028–4033 (2010). 20160114 10.1073/pnas.1000315107 PMC2840151

[b21] Trotter E. W., Rand J. D., Vickerstaff J. & Grant C. M. The yeast Tsa1 peroxiredoxin is a ribosome-associated antioxidant. Biochem J 412, 73–80 (2008). 18271751 10.1042/BJ20071634

[b22] Rand J. D. & Grant C. M. The Thioredoxin System Protects Ribosomes against Stress-induced Aggregation. Mol. Biol. Cell 17, 387–401 (2006). 16251355 10.1091/mbc.E05-06-0520 PMC1345676

[b23] Ruepp A. . The FunCat, a functional annotation scheme for systematic classification of proteins from whole genomes. Nucleic Acids Res 32, 5539–5545 (2004). 15486203 10.1093/nar/gkh894 PMC524302

[b24] Washburn M. P., Wolters D. & Yates J. R. R. Large-scale analysis of the yeast proteome by multidimensional protein identification technology. Nat. Biotechnol. 19, 242–247 (2001). 11231557 10.1038/85686

[b25] Belle A., Tanay A., Bitincka L., Shamir R. & O’Shea E. K. Quantification of protein half-lives in the budding yeast proteome. Proc Natl Acad Sci USA 103, 13004–13009 (2006). 16916930 10.1073/pnas.0605420103 PMC1550773

[b26] Michelitsch M. D. & Weissman J. S. A census of glutamine/asparagine-rich regions: implications for their conserved function and the prediction of novel prions. Proc Natl Acad Sci USA 97, 11910–11915 (2000). 11050225 10.1073/pnas.97.22.11910 PMC17268

[b27] Arava Y. . Genome-wide analysis of mRNA translation profiles in Saccharomyces cerevisiae. Proc Natl Acad Sci USA 100, 3889–3894 (2003). 12660367 10.1073/pnas.0635171100 PMC153018

[b28] Willmund F. . The cotranslational function of ribosome-associated Hsp70 in eukaryotic protein homeostasis. Cell 152, 196–209 (2013). 23332755 10.1016/j.cell.2012.12.001 PMC3553497

[b29] Gong Y. . An atlas of chaperone-protein interactions in Saccharomyces cerevisiae: implications to protein folding pathways in the cell. Mol Syst Biol 5, 275 (2009). 19536198 10.1038/msb.2009.26 PMC2710862

[b30] Pausch P. . Co-translational capturing of nascent ribosomal proteins by their dedicated chaperones. Nat Commun 6, 7494 (2015). 26112308 10.1038/ncomms8494 PMC4491177

[b31] Peters T. W. . Tor1 regulates protein solubility in Saccharomyces cerevisiae. Mol Biol Cell 23, 4679–4688 (2012). 23097491 10.1091/mbc.E12-08-0620 PMC3521677

[b32] David D. C. . Widespread protein aggregation as an inherent part of aging in *C. elegans*. PLoS Biol 8, e1000450 (2010). 20711477 10.1371/journal.pbio.1000450 PMC2919420

[b33] Castillo V., Grana-Montes R. & Ventura S. The aggregation properties of Escherichia coli proteins associated with their cellular abundance. Biotechnol J 6, 752–760 (2011). 21538899 10.1002/biot.201100014

[b34] Bednarska N. G., Schymkowitz J., Rousseau F. & Van Eldere J. Protein aggregation in bacteria: the thin boundary between functionality and toxicity. Microbiology 159, 1795–1806 (2013). 23894132 10.1099/mic.0.069575-0

[b35] Tartaglia G. G., Pechmann S., Dobson C. M. & Vendruscolo M. Life on the edge: a link between gene expression levels and aggregation rates of human proteins. Trends Biochem Sci 32, 204–206 (2007). 17419062 10.1016/j.tibs.2007.03.005

[b36] Gsponer J. & Babu M. M. Cellular strategies for regulating functional and nonfunctional protein aggregation. Cell Rep 2, 1425–1437 (2012). 23168257 10.1016/j.celrep.2012.09.036 PMC3607227

[b37] Ciryam P., Tartaglia G. G., Morimoto R. I., Dobson C. M. & Vendruscolo M. Widespread aggregation and neurodegenerative diseases are associated with supersaturated proteins. Cell Rep 5, 781–790 (2013). 24183671 10.1016/j.celrep.2013.09.043 PMC3883113

[b38] Reis-Rodrigues P. . Proteomic analysis of age-dependent changes in protein solubility identifies genes that modulate lifespan. Aging cell 11, 120–127 (2012). 22103665 10.1111/j.1474-9726.2011.00765.x PMC3437485

[b39] Grant C. M. Regulation of translation by hydrogen peroxide. Antioxid Redox Signal 15, 191–203 (2011). 21126188 10.1089/ars.2010.3699

[b40] Pechmann S., Levy E. D., Tartaglia G. G. & Vendruscolo M. Physicochemical principles that regulate the competition between functional and dysfunctional association of proteins. Proc Natl Acad Sci USA 106, 10159–10164 (2009). 19502422 10.1073/pnas.0812414106 PMC2700930

[b41] Rousseau F., Serrano L. & Schymkowitz J. W. How evolutionary pressure against protein aggregation shaped chaperone specificity. J Mol Biol 355, 1037–1047 (2006). 16359707 10.1016/j.jmb.2005.11.035

[b42] David D. C. Aging and the aggregating proteome. Frontiers in genetics 3, 247 (2012). 23181070 10.3389/fgene.2012.00247 PMC3501694

[b43] Ghaemmaghami S. . Global analysis of protein expression in yeast. Nature 425, 737–741 (2003). 14562106 10.1038/nature02046

